# Differences in the choroid plexus volume and microstructure are associated with body adiposity

**DOI:** 10.3389/fendo.2022.984929

**Published:** 2022-10-13

**Authors:** Joseph S. R. Alisch, Josephine M. Egan, Mustapha Bouhrara

**Affiliations:** Laboratory of Clinical Investigation, National Institute on Aging, National Institutes of Health, Baltimore, MD, United States

**Keywords:** choroid plexus, body mass index, waist circumference, magnetic resonance imaging, obesity

## Abstract

The choroid plexus (CP) is a cerebral structure located in the ventricles that functions in producing most of the brain’s cerebrospinal fluid (CSF) and transporting proteins and immune cells. Alterations in CP structure and function has been implicated in several pathologies including aging, multiple sclerosis, Alzheimer’s disease, and stroke. However, identification of changes in the CP remains poorly characterized in obesity, one of the main risk factors of neurodegeneration, including in the absence of frank central nervous system alterations. Our goal here was to characterize the association between obesity, measured by the body mass index (BMI) or waist circumference (WC) metrics, and CP microstructure and volume, assessed using advanced magnetic resonance imaging (MRI) methodology. This cross-sectional study was performed in the clinical unit of the National Institute on Aging and included a participant population of 123 cognitively unimpaired individuals spanning the age range of 22 – 94 years. Automated segmentation methods from FreeSurfer were used to identify the CP structure. Our analysis included volumetric measurements, quantitative relaxometry measures (*T*_1_ and *T*_2_), and the diffusion tensor imaging (DTI) measure of mean diffusivity (MD). Strong positive associations were observed between WC and all MRI metrics, as well as CP volume. When comparing groups based on the established cutoff point by the National Institutes of Health for WC, a modest difference in MD and a significant difference in *T*_1_ values were observed between obese and lean individuals. We also found differences in T1 and MD between obese and overweight individuals as defined using the BMI cutoff. We conjecture that these observations in CP volume and microstructure are due to obesity-induced inflammation, diet, or, very likely, dysregulations in leptin binding and transport. These findings demonstrate that obesity is strongly associated with a decline in CP microstructural integrity. We expect that this work will lay the foundation for further investigations on obesity-induced alterations in CP structure and function.

## Introduction

Obesity, which is characterized by excessive adipose tissue, is a complex disease that impacts several physiological systems of the body. It also renders people at increased risk for an array of other diseases including, but not limited to, diabetes, cardiovascular diseases and cancer—all of which drastically increase the global health burden of this condition (1, 2). Many lines of research have also provided support for an association between obesity and several central nervous system (CNS) pathologies such as Alzheimer’s disease (3, 4) and multiple sclerosis (5, 6). Obesity is understood to enact a variety of down-stream effects in the CNS through decreased myelin content (7), and effects on cortical thickness (8), gray matter volume (9) and cerebral blood flow (10). Further, a common feature for many of these neurodegenerative processes is inflammation, which is attenuated by exercise and, not surprisingly, dietary interventions (11, 12).

Implicated in mediating the neuroinflammatory effects of obesity is the choroid plexus (CP) (13–15), a critical cerebral structure necessary for cerebrospinal fluid (CSF) production. The CP is housed in the ventricles, which are a network of four communicating cavities responsible for providing protection and circulating CSF to the brain. Structurally, the CP consists of a single layer of cuboidal epithelial cells connected by apical tight junctions that interfaces CSF and fenestrated capillaries forming the blood-CSF barrier (BCSFB) (16). Moreover, the CP has various other functions such as strict regulation of transport systems that modulate CSF composition by allowing passage of nutrients such as glucose, lipids and proteins, solutes and inflammatory cells (16), as well as producing growth factors and hormones involved with development and maintenance of brain function (17). Previous research has shown that dysfunction of the CP and ventriculomegaly are involved in the pathogenesis of neurodegenerative diseases including Alzheimer’s disease (18–20) and multiple sclerosis (21–23). Additionally, diet formulations in studies that contain high fat and cholesterol rich content have been suggested to contribute to these pathological features (14, 24, 25).

Several methods used in identifying CP structure and lateral ventricles (LV) include magnetic resonance imaging (MRI) quantitative techniques (26). Current works that have evaluated the CP *in-vivo* used volumetric analyses, relaxometry, specifically longitudinal and transverse relaxation times (*T*_1_ and *T*_2_), and mean diffusivity (MD) derived from diffusion tensor imaging (DTI), to assess CP volume and microstructure (27–32). *T*_1_ and *T*_2_ both depend on macromolecular tissue composition as well as water mobility. Thus, observed changes in *T*_1_ or *T*_2_ are directly associated with cerebral microstructural tissue changes (33). Similarly, MD can also be used to identify cerebral tissue microstructural integrity based on water content and mobility (34, 35). These previous MRI-based studies have shown that the CP and LV significantly increase in volume during disease states and normative aging (28–32). Moreover, the CP exhibits increases in *T*_1_, *T*_2_ and MD during normative aging, reflecting increased water mobility that suggests a decrease in microstructural integrity (27, 28). In a cross-sectional study that looked at obesity in Alzheimer’s disease patients, Ho and colleagues found that after performing a *T*_1_-weighted imaging volumetric analysis, cerebral ventricular enlargement was associated with a high body mass index (BMI) after controlling for age, sex, and education (36). The authors conjectured that a high BMI could result in different outcomes in brain volume measurements. In another study that looked at the relationship between allostatic load and CP volume that was measured from *T*_1_-weighted imaging, the authors found a positive association in individuals with schizophrenia (37). Briefly, allostatic load represents the culmination of chronic stress and is a combined measure of cardiovascular indicators, metabolic indicators including BMI and waist circumference (WC), inflammation, and neuroendocrine hormones (37). While not specific to obesity, previous studies have shown an association between obesity and high allostatic load due to several factors such as diet, systemic inflammation, and other lifestyle and environmental characteristics (38). Despite the current available literature, no MRI study, to our knowledge, has examined the microstructural impact of obesity on the CP in the absence of a major CNS pathology.

In this study, our participant population included 123 cognitively unimpaired subjects with healthy weight, overweight, or obesity over the age range of 22 – 94 years. Our main goal was to elucidate the relationship between LV volume, CP volume, and CP microstructure, which was assessed using *T*_1_, *T*_2_ or MD, and BMI or WC, as measures of obesity. Here, we lay the groundwork in our understanding of CP and LV-specific changes related to metabolic dysfunction, specially, obesity.

## Materials and methods

### Participants

Experimental procedures were performed in compliance with our local Institutional Review Board, and participants provided written informed consent. Participants were drawn from the Baltimore Longitudinal Study of Aging (BLSA) (39, 40), and the Genetic and Epigenetic Signatures of Translational Aging Laboratory Testing (GESTALT) study. The study populations, experimental design, and measurement protocols of the BLSA have been previously reported (39, 40). The goal of the BLSA and GESTALT studies is to evaluate multiple biomarkers related to aging. We note that the inclusion and exclusion criteria for these two studies are essentially identical. Participants underwent testing at the National Institute on Aging’s clinical research unit and were excluded if they had metallic implants, neurologic, or medical disorders. Further, all participants underwent a battery of cognitive tests and participants with cognitive impairment were excluded (41).

### MR imaging

We emphasize that all MRI studies and ancillary measurements were performed with the same MRI system, running the same pulse sequences, at the same facility, and directed by the same investigators for both BLSA and GESTALT participants. The MRI protocol was approved by the MedStar Research Institute and the National Institutes of Health Intramural Ethics Committees, and all examinations were performed in compliance with the standards established by the National Institutes of Health Institutional Review Board. MRI scans were performed on a 3T whole body Philips MRI system (Achieva, Best, The Netherlands) using the internal quadrature body coil for transmission and an eight-channel phased-array head coil for reception. For each participant, the *T_1_* and *T_2_* maps were derived from 3D spoiled gradient recalled echo (SPGR) images were acquired with flip angles of [2 4 6 8 10 12 14 16 18 20]°, echo time (TE) of 1.37 ms, repetition time (TR) of 5 ms, and acquisition time of ~5 min, as well as 3D balanced steady state free precession (bSSFP) images acquired with flip angles of [2 4 7 11 16 24 32 40 50 60]°, TE of 2.8 ms, TR of 5.8 ms, and acquisition time of ~6 min. The bSSFP images were acquired with radiofrequency excitation pulse phase increments of 0 or π in order to account for off-resonance effects (42–45). All SPGR and bSSFP images were acquired with an acquisition matrix of 150 × 130 × 94, voxel size of 1.6 mm × 1.6 mm × 1.6 mm. Further, we used the double-angle method (DAM) to correct for excitation radio frequency inhomogeneity (46). For this, two fast spin-echo images were acquired with flip angles of 45° and 90°, TE of 102 ms, TR of 3000 ms, acquisition voxel size of 2.6 mm × 2.6 mm × 4 mm, and acquisition time of ~4 min. All images were acquired with field of view (FoV) of 240 mm × 208 mm × 150 mm. The total acquisition time was ~21 min. Finally, the MD map was derived from the DTI dataset. The DTI protocol consisted of diffusion-weighted images (DWI) acquired with single-shot EPI, TR of 10,000 ms, TE of 70 ms, two *b*-values of 0 and 700 s/mm^2^, with the latter encoded in 32 directions, acquisition matrix of 120 × 104 × 75, and acquisition voxel size of 2 mm × 2 mm × 2 mm. Images were acquired with FoV of 240 mm × 208 mm × 150 mm. All images were reconstructed to a voxel size of 1 mm × 1 mm × 1 mm.

### Image processing

**Figure 1:**
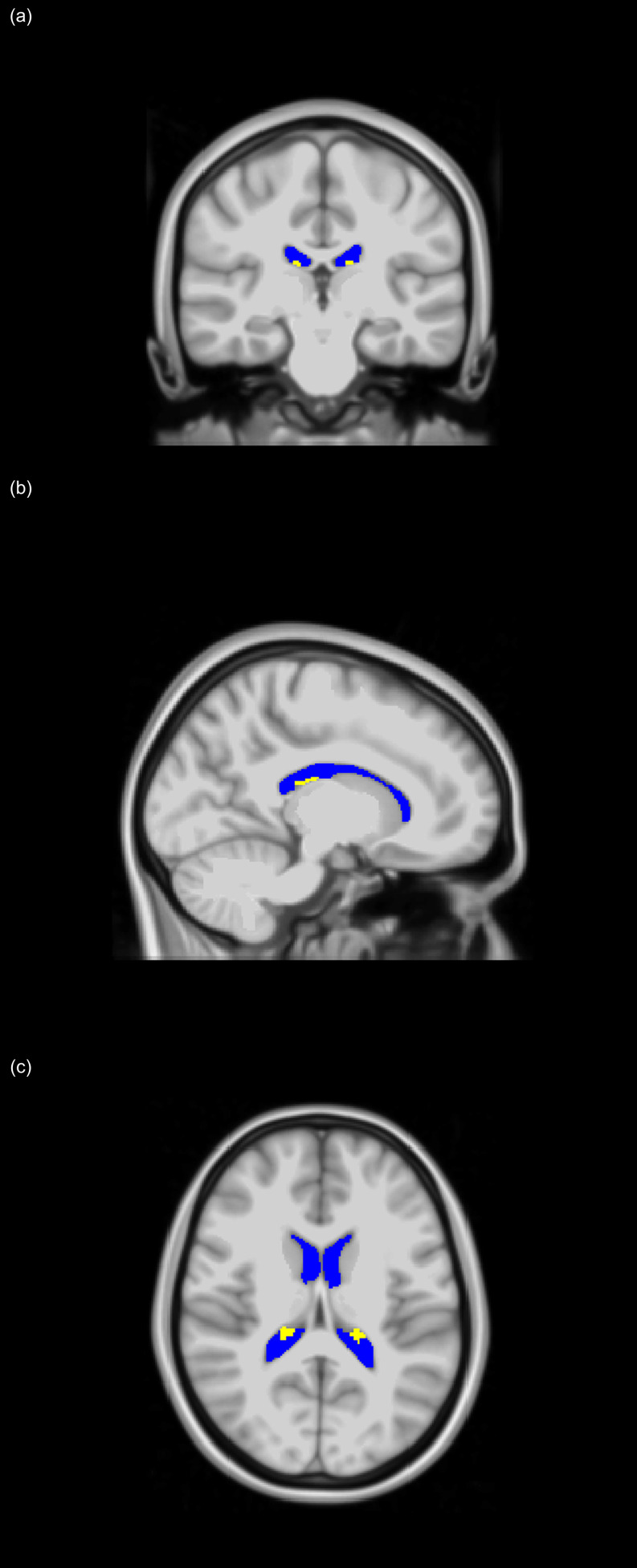
Choroid plexus (CP - yellow) and lateral ventricles (LV - blue) regions of interest (ROIs), extracted using FreeSurfer and projected on the MNI atlas for three different views: **(A)** coronal, **(B)** sagittal, and **(C)** axial. All images are in radiological convention.

CP and LV volumes calculation: for each participant, corresponding *T_1_*-weighted SPGR images were used. Specifically, the FreeSurfer Aseg Atlas (47) was nonlinearly registered to the SPGR images averaged over all flip angles using the cortical reconstruction (*recon-all*) pipeline from the Freesurfer v7.1.1 software^1^ (48) (**Figure 1**). Volumetric measurements were then extracted from the CP and LV regions of interest (ROIs). This method has been used in several other studies indicating reliable CP and LV segmentation (28–30, 32, 34, 49, 50). All CP and LV ROIs were thoroughly examined and corrected manually when needed.

T_1_ and T_2_ mapping: using the FMRIB Software Library (FSL) software (51), all SPGR, bSSFP, and DAM images were linearly registered to the SPGR image acquired at a flip angle of 8° and the derived transformation matrix was then applied to the original SPGR, bSSFP, and DAM images. Next, a whole-brain T_1_ map was generated from the co-registered SPGR dataset using the DESPOT1 analysis and assuming a single relaxing component using the stochastic regions contraction (SRC) algorithm while correcting for transmit field, B_1_, inhomogeneities (52, 53). The B_1_ map was generated from the co-registered fast spin-echo using the DAM approach (46). Further, using these derived T_1_ and B_1_ maps as input parameters, a whole-brain T_2_ map was generated from the co-registered bSSFP dataset using the DESPOT2 analysis and assuming a single component using the SRC algorithm (52, 53). *B_1_*, *T_1_*, and *T_2_* maps were generated using in-house MATLAB scripts. All these MATLAB codes are available upon request. Next, using FreeSurfer, the SPGR image averaged over all flip angles for each participant was registered using nonlinear registration to FreeSurfer’s Aseg atlas and the derived transformation matrix was then applied to the corresponding T_1_ and T_2_ maps. Finally, the mean T_1_ and T_2_ values in the CP ROI were calculated. We note that 10 datasets were excluded from the subsequent analyses due to issues with the imaging data including motion artefacts.

MD mapping: the DW images were corrected for eddy current and motion effects using affine registration tools implemented in FSL (51), and registered to the DW image obtained with *b* = 0 s/mm^2^. Moreover, whole-brain MD map was derived from the co-registered DWI data. We used the *DTIfit* tool implemented in FSL to calculate the eigenvalue maps which were used to calculate MD (54). Finally, for each participant, the DW image obtained at *b* = 0 s/mm^2^ was nonlinearly registered to FreeSurfer’s Aseg atlas and the calculated matrix of transformation was then applied to the corresponding MD map. Finally, the mean MD value in the CP ROI was calculated. We note that 8 datasets were excluded from the subsequent analyses due to issues with the imaging data including motion artefacts.

### Statistical analysis

For each ROI, the correlation between BMI or WC and CP volume, LV volume, *T_1_*, *T_2_*, or MD was investigated using multiple linear regression with the mean CP volume, LV volume, *T_1_*, *T_2_*, or MD value within the ROI as the dependent variable and BMI or WC, sex, ethnicity, age, and age^2^ as independent variables, after mean age centering. The inclusion of age^2^ as an independent variable is based on our and others’ recent observations that cerebral maturation follows a quadratic relationship with age (55, 56). We note that the CP and LV volumes were corrected for the total intracranial volume (ICV). Further, given the potential correlation between the CP volume and the LV volume, the association between the CP volume and BMI or WC was also conducted while accounting for the LV volume.

To investigate the associations between BMI or WC and CP volume, LV volume, *T_1_*, *T_2_*, or MD between the groups of subjects with healthy weight (lean), overweight, and obesity, we performed between-group ANCOVA analyses for each ROI. These included i) obese vs. lean groups, ii) overweight vs. lean groups, and iii) obese vs. overweight groups. All between-group comparisons controlled for sex, ethnicity, age and age^2^.

Finally, to explore the potential effect of partial volume on the association between *T_1_*, *T_2_*, or MD and BMI or WC due to contamination from CSF, we calculated mean values of these MR parameters in the ventricular CSF regions and conducted a regression analysis accounting for the confounding variable as described above. CSF maps were generated using FSL FAST segmentation (57).

The threshold for statistical significance was set to *p* < 0.05. Given the exploratory nature of this study and concerns about type 2 error, all analyses were conducted without correction for multiple comparison. Calculations were performed with MATLAB (MathWorks, Natick, MA, USA). All MATLAB codes are available upon request from the corresponding author.

## Results

The final cohort consisted of 123 cognitively unimpaired volunteers ranging in age from 21 to 94 years (55.0 ± 20.5 years) of which 65 were men (56.0 ± 21.5 years) and 58 were women (54.0 ± 19.6 years), after exclusion of nine participants with cognitive impairment (**Table 1**). Age (*p* > 0.1) did not differ significantly between men and women. The number of participants per age-decade was: 14 (7 females) within 20-29 years, 17 (6 females) within 30-39 years, 35 (20 females) within 40-49 years, 8 (3 females) within 50-59 years, 9 (6 females) within 60-69 years, 17 (7 females) within 70-79 years, 21 (9 females) within 80-89 years, and 2 (0 females) within 90-99 years. Following established BMI cutoff points (58), the cohort consisted of 56 lean participants (BMI < 25), 50 overweight participants (25 ≤ BMI < 30), and 17 participants with obesity (BMI ≥ 30), while following the National Institutes of Health cutoff points for WC, the cohort consisted of 71 lean participants (WC < 94 cm for men and WC < 80 cm for women), 31 overweight participants (94 ≤ WC < 102 for men and 80 ≤ WC < 88 for women), and 21 participants with obesity (WC ≥ 102 for men and WC ≥ 88 for women). Finally, we note that BMI and WC exhibited a modest correlation leading to a coefficient of correlation value of *R^2^* = 0.54.

**Table 1:** Demographic characteristics of participants.

Participant Demographics	Total Sample (*N* = 123)
**Age (yrs.): mean ± SD *(min – max)***	56.2 ± 20.9 *(22-94)*
**Sex**	
Men	65 *(52.8%)*
Women	58 *(47.2%)*
**MMSE**	28.8 ± 1.4 *(25-30)*
**BMI (Kg/m^2^): mean ± SD *(min – max)***	25.8 ± 3.6 *(18.3-35.8)*
**WC (cm): mean ± SD *(min – max)***	86.9 ± 10.4 *(63.0-112.8)*

SD, standard deviation; min, minimum; max, maximum; MMSE, Mini‐Mental State Examination; BMI, body-mass index; WC, waist circumference. Italic indicates the percentage or range of the variable.

**Figure 2:**
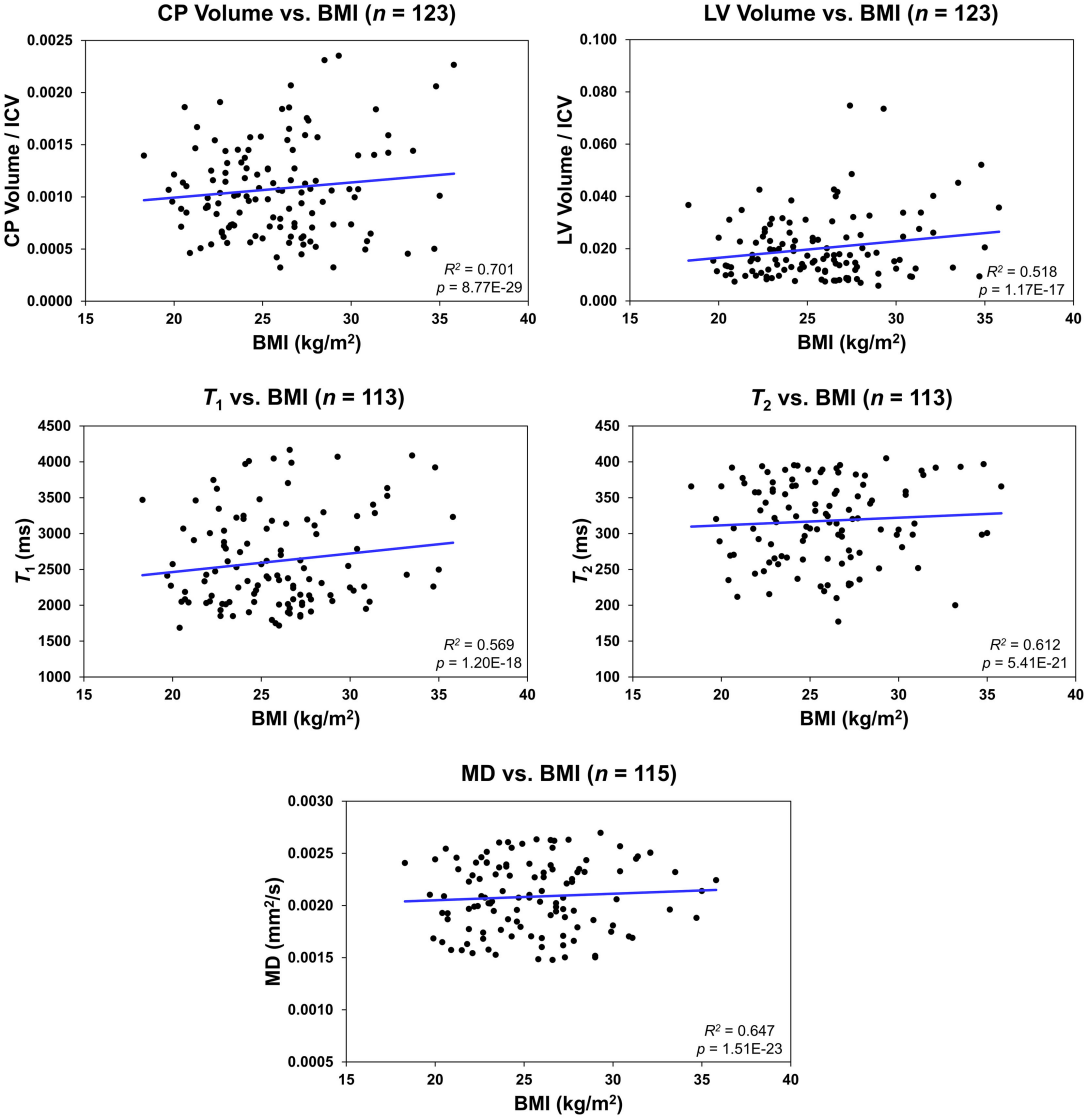
Regression results for the relationship between the lateral ventricle (LV) volume/intracranial volume (ICV), *T_1_*, *T_2_*, or mean diffusivity (MD) and body mass index (BMI) adjusted for age, age^2^, sex, and ethnicity. For the choroid plexus (CP) volume/ICV, regression results were controlled for age, age^2^, sex, ethnicity, and LV volume. The coefficient of determination, *R^2^*, and p-value, *p*, of the multiple linear regression model are reported. The CP volume, LV volume, *T_1_*, *T_2_*, or MD exhibited significant positive correlations with BMI.

**Figure 3:**
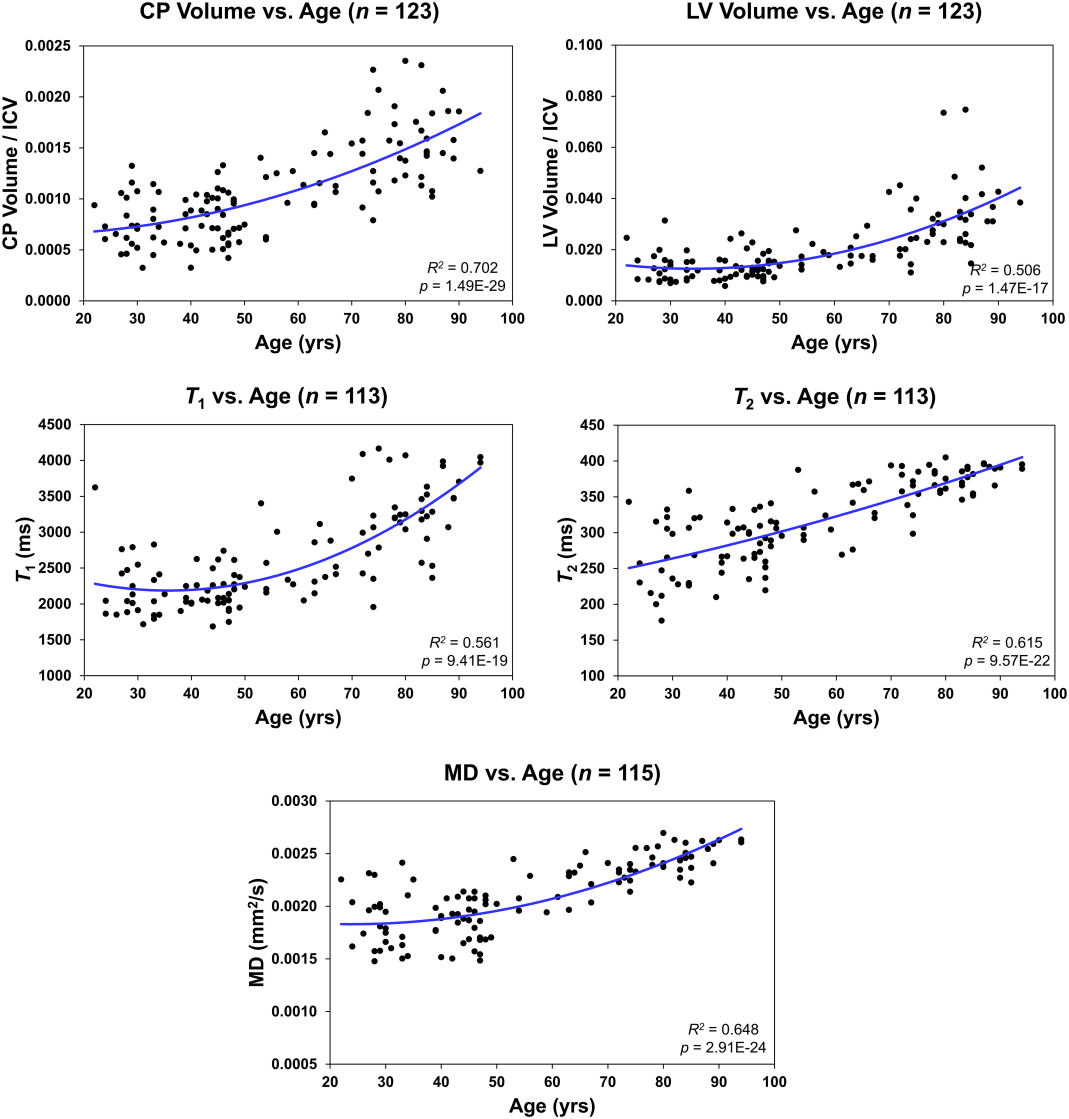
Regressions of choroid plexus (CP) volume/intracranial volume (ICV), lateral ventricle (LV) volume/ICV, *T_1_*, *T_2_*, or mean diffusivity (MD) with age. For each plot, the coefficient of determination, *R^2^*, and p-value, *p*, are reported.

**Figure 2** shows regression relationships between the CP volume, LV volume, after correcting for the total intracranial volume, *T_1_*, *T_2_*, or MD and BMI, after adjusting for sex, ethnicity, age, and age^2^. Visual inspection indicates that higher BMI values corresponds to higher CP volume, LV volume, *T_1_*, *T_2_*, or MD values. Accounting for the LV volume in the regression of CP vs BMI or WC had a marginal effect. Further, the multiple regression analysis indicates that the correlation between the LV volume or *T_1_* and BMI was statistically significant (*p_BMI_* < 0.05) or close to significance (*p_BMI_* < 0.1) (**Figure 2**; **Table 2**). Furthermore, as expected, a statistically significant age effect was found for all MR metrics evaluated (**Figure 3**; **Table 2**). Similarly, the quadratic effect of age, age^2^, was statistically significant or close to significance for most metrics (**Figure 3**; **Table 2**). Additionally, the correlation between CP volume or MD and sex was found to be statistically significant (**Table 2**).

**Table 2:** Slope, *β*, and significance, *p*, of the regression terms incorporated in the multiple linear regression given by: Lateral ventricle (LV) volume/intracranial volume (ICV), *T_1_*, *T_2_*, or mean diffusivity (MD) ~ *β_0_* + *β_BMI_* × BMI + *β_age_* × age + *β_age_^2^* ×age^2^ + *β_sex_* × sex + *β_ethnicity_* × ethnicity.

	Age		Age^2^		Sex		Ethnicity		BMI	
	*β*_age_	*p*_age_	*β*_age_^2^	*p*_age_^2^	*β*_sex_	*p*_sex_	*β*_ethnicity_	*p*_ethnicity_	*β*_BMI_	*p*_BMI_
CP volume/ICV	7.39 x 10^-6^	**2.56 x 10^-6^**	-4.99 x 10^-8^	0.49	1.01 x 10^-4^	**0.03**	-2.84 x 10^-5^	0.70	-4.28 x 10^-6^	0.51
LV volume/ICV	3.56 x 10^-4^	**1.82 x 10^-14^**	9.17 x 10^-6^	**1.75 x 10^-4^**	1.45 x 10^-4^	0.93	-1.51 x 10^-3^	0.55	4.43 x 10^-4^	**4.77 x 10^-2^**
*T_1_*	20.20	**6.63 x 10^-17^**	0.52	**2.54 x 10^-5^**	18.21	0.83	-61.85	0.66	20.25	**0.07**
*T_2_*	2.08	**1.46 x 10^-22^**	9.29 x 10^-3^	0.34	-2.43	0.72	-16.69	0.15	0.48	0.61
MD	1.13 x 10^-5^	**9.67 x 10^-21^**	1.57 x 10^-7^	**4.82 x 10^-3^**	1.02 x 10^-4^	**8.93 x 10^-3^**	2.10 x 10^-6^	0.97	4.09 x 10^-6^	0.45

For the choroid plexus (CP) volume, the multiple linear regression model includes the LV volume as an additional covariate given by CP volume/ICV ~ *β_0_* + *β_BMI_* × BMI + *β_age_* × age + *β_age_^2^* ×age^2^ + *β_sex_* × sex + *β_ethnicity_* × ethnicity + *β_LV_* × LV. Sex and ethnicity results are not shown for associations with CP volume, LV volume, *T_1_*, *T_2_*, or MD. Bold indicates significance (p < 0.05) or close to significance (p < 0.1).

**Figure 4:**
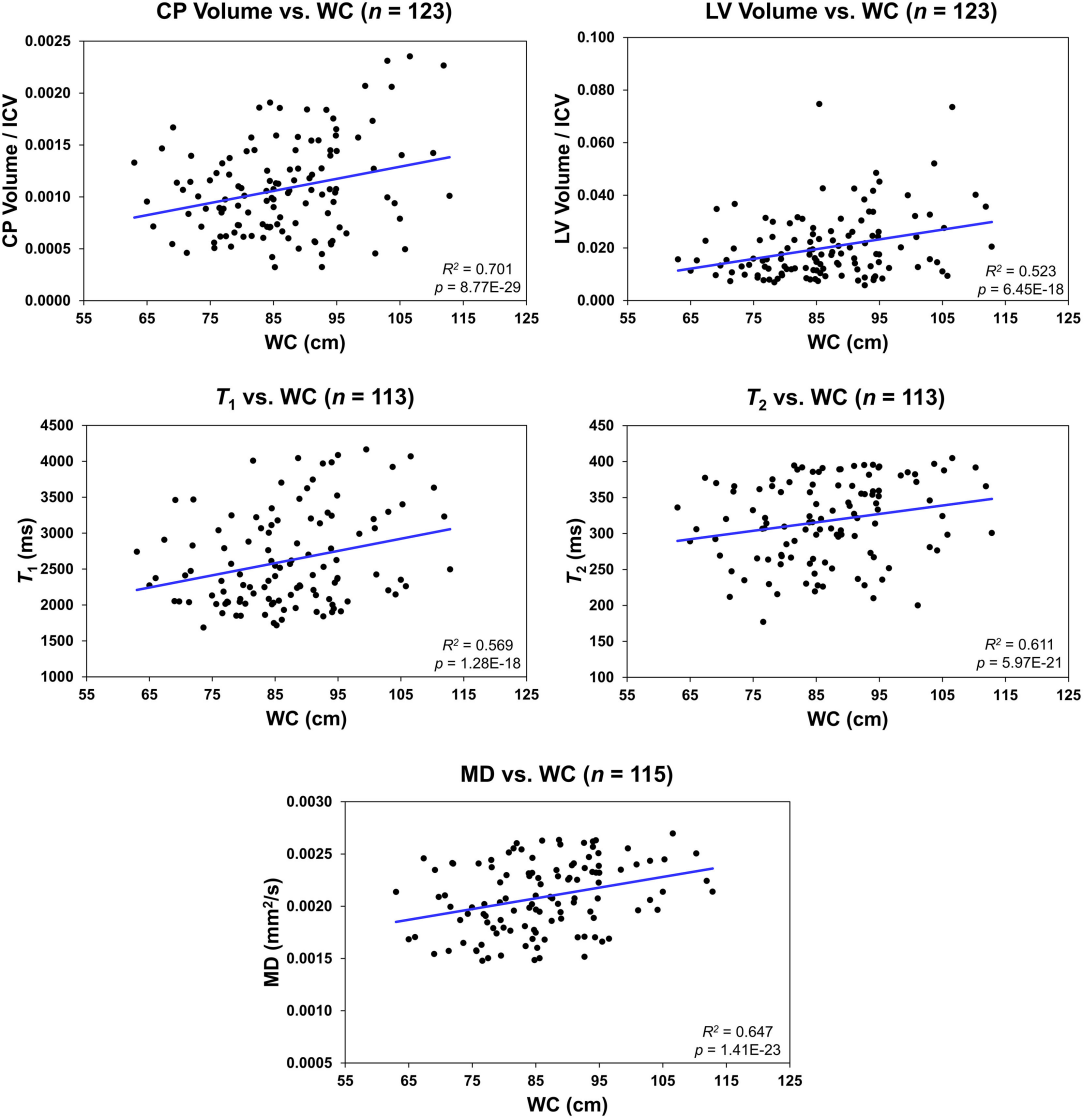
Regression results for the relationship between the lateral ventricle (LV) volume/intracranial volume (ICV), *T_1_*, *T_2_*, or mean diffusivity (MD) and waist circumference (WC) adjusted for age, age^2^, sex, and ethnicity. For the choroid plexus (CP) volume/ICV, regression results were controlled for age, age^2^, sex, ethnicity, and LV volume. The coefficient of determination, *R^2^*, and p-value, *p*, of the multiple linear regression model are reported. The CP volume, LV volume, *T_1_*, *T_2_*, or MD exhibited significant positive correlations with WC.

**Figure 4** shows regression relationships between the CP volume, LV volume, *T_1_*, *T_2_*, or MD and WC, after adjusting for sex, ethnicity, age, and age^2^. Visual inspection indicates that larger WC corresponds to higher CP volume, LV volume, *T_1_*, *T_2_*, or MD values. Further, the multiple regression analysis indicates that this correlation between the LV volume or *T_1_* and WC was statistically significant (*p_WC_* < 0.05) or close to significance (*p_WC_* < 0.1) (**Table 3**). Furthermore, as expected, a statistically significant age effect was found for all metrics evaluated (**Table 3**). Moreover, the quadratic effect of age, age^2^, was statistically significant for most MR metrics (**Table 3**). Additionally, the correlation between CP volume or MD and sex was found to be statistically significant (**Table 3**).

**Table 3:** Slope, *β*, and significance, *p*, of the regression terms incorporated in the multiple linear regression given by: Lateral ventricle (LV) volume/intracranial volume (ICV), *T_1_*, *T_2_*, or mean diffusivity (MD) ~ *β_0_* + *β_WC_* × WC + *β_age_* × age + *β_age_^2^* ×age^2^ + *β_sex_* × sex + *β_ethnicity_* × ethnicity.

	Age		Age^2^		Sex		Ethnicity		WC	
	*β*_age_	*p*_age_	*β*_age_^2^	*p*_age_^2^	*β*_sex_	*p*_sex_	*β*_ethnicity_	*p*_ethnicity_	*β*_WC_	*p*_WC_
CP volume/ICV	7.79 x 10^-6^	**8.45 x 10^-7^**	-7.06 x 10^-8^	0.33	1.40 x 10^-4^	**8.39 x 10^-3^**	-2.58 x 10^-5^	0.72	-4.19 x 10^-6^	0.12
LV volume/ICV	3.30 x 10^-4^	**4.86 x 10^-12^**	9.84 x 10^-6^	**6.86 x 10^-5^**	-1.65 x 10^-3^	0.37	-1.73 x 10^-3^	0.49	2.10 x 10^-4^	**2.32 x 10^-2^**
*T_1_*	19.34	**6.12 x 10^-15^**	0.54	**1.65 x 10^-5^**	-45.87	0.63	-63.61	0.65	8.06	**0.06**
*T_2_*	2.08	**3.65 x 10^-21^**	9.32 x 10^-3^	0.35	-2.96	0.71	-16.68	0.15	0.09	0.81
MD	1.11 x 10^-5^	**7.12 x 10^-19^**	1.63 x 10^-7^	**4.03 x 10^-3^**	8.58 x 10^-5^	**0.05**	-2.48 x 10^-7^	0.99	1.88 x 10^-6^	0.39

For the choroid plexus (CP) volume, multiple linear regression model was given by CP volume/ICV ~ *β_0_* + *β_WC_* × WC + *β_age_* × age + *β_age_^2^* ×age^2^ + *β_sex_* × sex + *β_ethnicity_* × ethnicity + *β_LV_* × LV. Sex and ethnicity results are not shown for associations with CP volume, LV volume, *T_1_*, *T_2_*, or MD. Bold indicates significance (p < 0.05) or close to significance (p < 0.1).

**Table 4** shows demographic characteristics of the lean, overweight and obese subgroups stratified using the established BMI or WC cutoff points. While using the BMI cutoff point both the lean and overweight group incorporated a similar number of participants, the number of overweight participants decreased substantially when the WC cutoff point is used. In all case, the obese groups incorporated a much lower number of participants. There were no statistically significant differences in MMSE between groups for both cutoff points. **Table 5** summarizes the results of the between-group ANCOVA analyses of the associations between BMI or WC and CP volume, LV volume, *T_1_*, *T_2_*, or MD. In comparing subjects with obesity to lean subjects, we found higher CP volume, LV volume, *T_1_*, *T_2_* or MD in the group of subjects with obesity despite controlling for age, age^2^, sex, and ethnicity as well as LV volume for the CP volume analysis. This effect was significant for LV and T1,and close to statistical significance for MD. Moreover, controlling for these same covariates, while comparison of overweight to lean subjects as defined by BMI or WC did not show statistically significant differences in CP volume, LV volume, *T_1_*, *T_2_*, or MD, we found higher CP volume, LV volume, *T_1_*, *T_2_*, or MD in the overweight group as compared to the lean group. Finally, in comparing subjects with obesity to overweight subjects as defined by BMI, we found significantly higher *T_1_* and MD in the group of subjects with obesity, with both groups defined by either BMI or WC exhibiting higher CP volume, LV volume, *T_1_*, *T_2_*, or MD values in the subjects with obesity as compared to overweight subjects. The mean and standard deviation values of each MRI metric for each group are provided in **Table 6**.

**Table 4:** Demographic characteristics of each group stratified using the established body mass index (BMI) cutoff points for lean participants (BMI < 25), overweight participants (25 ≤ BMI < 30), and participants with obesity (BMI ≥ 30) or using the established waist circumference (WC) cutoff points for lean participants (WC < 94 cm for men and WC < 80 cm for women), overweight participants (94 ≤ WC < 102 for men and 80 ≤ WC < 88 for women), and participants with obesity (WC ≥ 102 for men and WC ≥ 88 for women).

	BMI			WC			
	*n* (M, W)	MMSE Mean ± SD	BMI (kg/m^2^) Mean ± SD	*n* (M, W)	MMSE Mean ± SD	Men (cm) Mean ± SD	Women (cm) Mean ± SD	
Lean	56 (25, 31)	28.73 ± 1.52	22.56 ± 1.52	71 (41,30)	28.76 ± 1.42	86.17 ± 4.62	73.68 ± 4.68	
Overweight	50 (32, 18)	28.85 ± 1.20	27.00 ± 1.15	31 (15,16)	28.93 ± 1.31	96.58 ± 2.72	84.10 ± 2.19	
Obese	17 (8, 9)	28.59 ± 2.09	32.22 ± 1.91	21 (9,12)	28.52 ± 1.94	105.38 ± 2.74	95.92 ± 7.54	

SD, standard deviation; MMSE, Mini‐Mental State Examination; BMI, body-mass index; WC, waist circumference; M, Men; W, Women.

**Table 5:** Significance, *p*-value, of the between-group ANCOVA analyses of the effect of body mass index (BMI) or waist circumference (WC) on lateral ventricle (LV) volume/intracranial volume (ICV), *T_1_*, *T_2_*, or mean diffusivity (MD).

	Obese vs. Lean			Overweight vs. Lean		Obese vs. Overweight	
	BMI		WC	BMI	WC	BMI	WC
CP volume/ICV		0.18	0.33	0.26	0.12	0.91	0.51
LV volume/ICV		**0.03**	**8.90 x10^-3^**	0.76	0.34	0.45	0.39
*T_1_*		0.12	**0.04**	0.37	0.48	**0.03**	0.54
*T_2_*		0.49	0.38	0.11	0.63	0.11	0.71
MD		0.39	**0.07**	0.12	0.73	**0.03**	0.75

All between-group comparisons were controlled for age, age^2^, sex, and ethnicity. For CP volume/ICV, between-group comparisons were controlled for age, age^2^, sex, ethnicity, and LV volume. Bold indicates significance (p < 0.05) or close to significance (p < 0.1).

**Table 6:** Mean and standard deviation (SD) values of each magnetic resonance imaging (MRI) metric. Each group was stratified using the established body mass index (BMI) cutoff points for lean participants (BMI < 25), overweight participants (25 ≤ BMI < 30), and participants with obesity (BMI ≥ 30) or using the established waist circumference (WC) cutoff points for lean participants (WC < 94 cm for men and WC < 80 cm for women), overweight participants (94 ≤ WC < 102 for men and 80 ≤ WC < 88 for women), and participants with obesity (WC ≥ 102 for men and WC ≥ 88 for women).

	BMI			WC		
	Lean	Overweight	Obese	Lean	Overweight	Obese
CP volume/ICV (10^-3^)	1.06 ± 0.35	1.06 ± 0.51	1.17 ± 0.57	1.01 ± 0.38	1.07 ± 0.46	1.31 ± 0.58
LV volume/ICV (10^-3^)	18.81 ± 8.85	19.94 ± 15.0	24.73 ± 13.6	17.33 ± 8.95	21.34 ± 14.35	27.56 ± 16.18
*T_1_* (ms)	2605 ± 614	2526 ± 665	2882 ± 696	2512 ± 604	2601 ± 687	2937 ± 674
*T_2_* (ms)	319 ± 54	309 ± 59	333 ± 57	309 ± 56	315 ± 63	346 ± 40
MD (mm^2^/s) (10^-3^)	2.08± 0.32	2.06± 0.36	2.18 ± 0.31	2.03± 0.33	2.11 ± 0.37	2.26 ± 0.26

**Figure 5:**
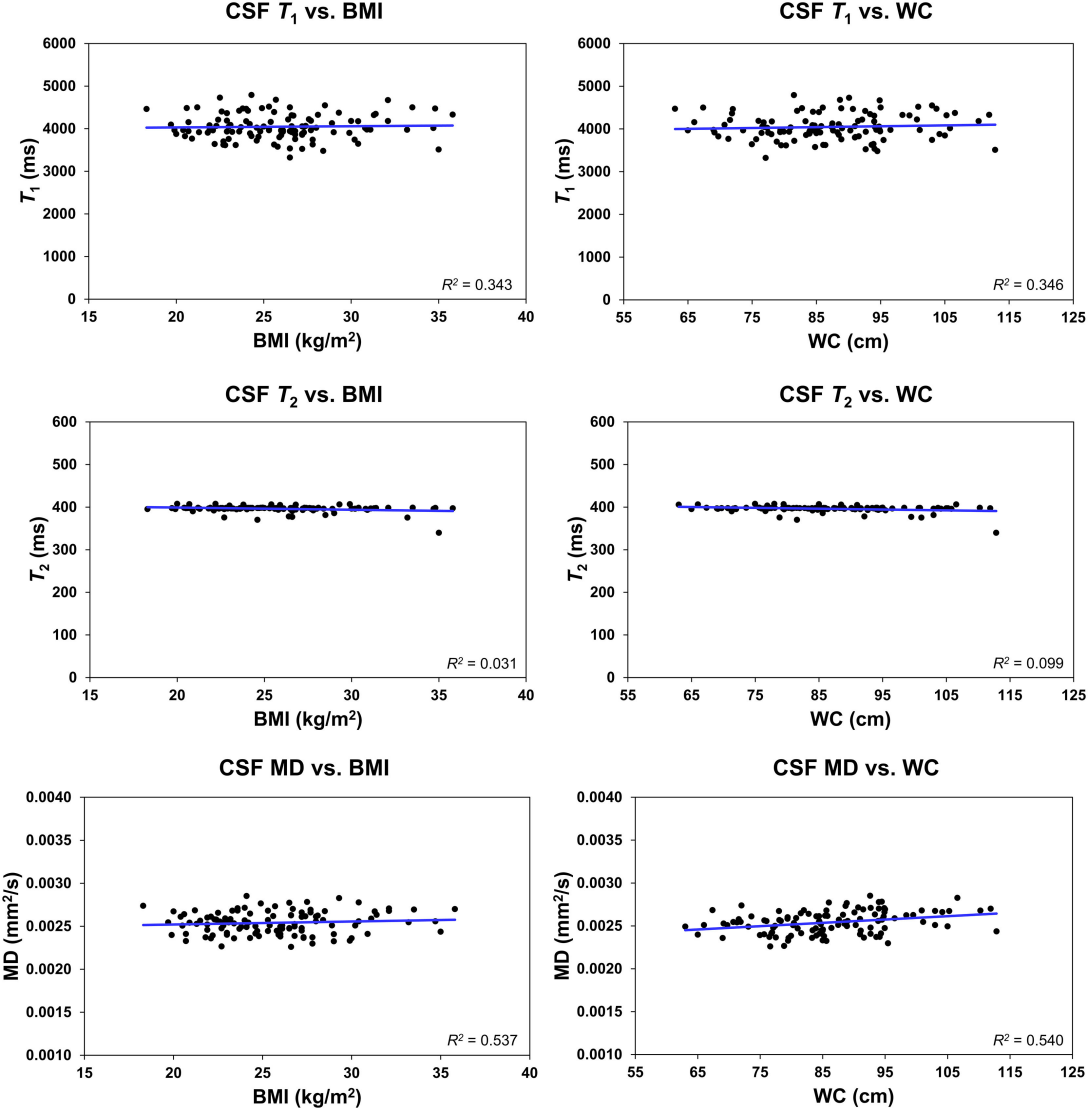
Regression results (*n* = 113) for the relationship between *T_1_*, *T_2_*, or mean diffusivity (MD), derived in the ventricular cerebrospinal fluid (CSF), and body mass index (BMI) or waist circumference (WC), adjusted for age, age^2^, sex, and ethnicity. The coefficient of determination, *R^2^*, is reported. All associations were statistically nonsignificant.

Further, it is readily seen that all MR parameters derived from the CSF exhibit a flat fitting line indicating no associations with BMI or WC (**Figure 5**). Our statistical analysis indicated that BMI and WC exhibited nonsignificant associations (*p* > 0.1) with each of the MR parameters of *T_1_*, *T_2_* and MD.

## Discussion

In this study, we provided the first report of an association between obesity and structural alterations in the CP using MR volumetry, quantitative relaxometry and DTI. When comparing groups of lean, overweight and obese stratified using the established cutoff points for WC (58), we found a modest difference in MD values and a significant difference in *T*_1_ values between obese and lean participants after correcting for age, sex and ethnicity, with MD and *T*_1_ exhibiting higher values with WC. Stratification by the BMI cutoff revealed significant differences in T1 and MD between obese and overweight individuals. Briefly, MD describes the overall diffusion and motion of water molecules in the brain (35, 59), while *T*_1_ describes the rate at which excited protons return to equilibrium and also depends on water content and mobility (33). For both of these MRI metrics, higher values reflect more water mobility or microstructural tissue deterioration. Therefore, the presence of increased water mobility in obese participants may be indicative of decreased microstructural integrity in the CP. We provisionally attribute this observation to dysregulations in leptin binding, which is a characteristic feature of obesity (60). Indeed, leptin is an adipocyte-derived hormone encoded by the obesity (*ob*) gene that regulates many physiological processes including energy homeostasis, appetite and immune function (61). The CP is one of the main access points by which leptin enters the CNS. Transport across the CP epithelium is regulated by the leptin receptors, which includes the highly expressed short form (Ob-Ra) and a long form (Ob-Rb), and megalin (LRP2) (62–64). In one study that examined the neuropathology of BTBR *ob/ob* mice, a model of leptin deficiency, the CP underwent ultrastructural remodeling, specifically atypical vacuolization and vesiculation of the basilar infoldings of the epithelial cells lining the LV. The authors concluded that this could represent a dysfunctional CP with abnormalities in leptin signaling (65).

In addition to ultrastructural remodeling, a compromised BCSFB has been implicated with obesity. One report showed that CSF/serum albumin ratio (QAlb), a biomarker of BCSFB permeability (66), was positively associated with both BMI and WC (67). A higher QAlb indicates higher permeability and is suggestive of damage to structures that function as barriers. Finally, in an animal study of diet-induced obesity, it has been found that consumption of a high energy diet decreased expression of tight junction proteins in the CP, thus compromising its structural integrity (14). However, increased water movement in the CP could also be due to increased cerebrospinal fluid secretion. Indeed, the CP secretes CSF and partially determines intracranial pressure (ICP). Mounting evidence, from preclinical and clinical investigations, have shown that obesity increases ICP which may lead to increased ventricular volume with obesity (68, 69), as observed here. Further histological investigations are required to shed the light on these aspects relating obesity to the CP’s microstructural differences.

Positive associations were also observed between obesity, measured either by BMI or WC, and CP volume, but did not have as strong of an effect as age. Previous research has shown that CP volume increases during pathological states such as stroke (30), multiple sclerosis (31) and Alzheimer’s disease (70), where neuro-inflammation is one of many characteristic features of these diseases. Obesity causes increasing inflammation in the CNS (15), as well as an increase in circulating serum leptin. In fact, one study found that serum leptin was 318% higher in individuals with obesity compared to lean participants. However, leptin concentration in the CSF of individuals with obesity was found to be only 30% higher than lean participants, which means a higher leptin CSF/serum ratio in lean participants (71). As mentioned previously, one of the roles of leptin is regulation of immune function. Leptin mediates recruitment of neutrophils at barrier structures, like the CP, involved in transporting immune cells into the brain (72). The increase in circulating leptin and, consequently, inflammation may serve as a potential explanation for the positive association since imaging studies on multiple sclerosis and depression have suggested a link between an increase in CNS inflammation and an enlarged CP (31, 73). However, the finding that BMI and WC did not have as a strong of an effect as age on CP volume suggests that they may not be as strong of an indicator for obesity-related changes in volume. Studies examining associations in obesity-induced inflammation and CP volume are encouraged.

Further, we found that the LV volume was significantly larger in individuals with obesity compared to lean participants when using WC to distinguish between groups. Additionally, WC was found to have a significant positive relationship with LV volume. This finding is consistent with previous studies with a larger patient population of individuals that found a significant association between BMI or WC and LV volume (36, 74).

Of note, BMI was not sensitive enough to capture the differences between obese and lean groups in our study. Besides differences in the number of subjects per group due to the BMI or WC stratifications, the BMI is not a robust surrogate for body fat mass, while high BMI does not necessarily result in a higher mortality (75, 76). In contrast, WC provides higher predictive power of disease risk than does BMI (77, 78). Indeed, increased WC correlated better with increased insulin resistance, cancer and dyslipidemia than does BMI due to its strong association with the amount of visceral fat (58, 79, 80). However, it has been shown that increased WC in men corresponds to increases visceral fat whereas in women tends to be more related to subcutaneous fat (81). Excess visceral fat is associated with more adverse cardio-metabolic side effects compared to excess subcutaneous fat, thus these factors could have independent effects on the outcomes in this. Further studies using DEXA scanning, the current gold-standard for determining regional body composition, are still required.

Although we examined a relatively large cohort and used advanced MR methodology, our study has limitations. Our dataset is cross-sectional so that the observed trends in the CP microstructure or volume with BMI or WC require further validation through longitudinal studies. Such work, motivated by the present results, is underway. Furthermore, our analysis of functional and structural differences in CP with aging were limited to the LVs. We note that CSF partial volume effects may bias derived parameter values (82). However, our analysis indicates that the observed trends of the MR parameters are driven by differences in the CP’s microstructure as all parameters derived in the CSF exhibited nonsignificant associations with BMI or WC. Nevertheless, more accurate automated segmentation methods, including the third and fourth ventricles, as well as higher resolution structural images are needed for a better evaluation. In addition, our DTI-related results must be interpreted with caution. Indeed, given the large fraction of free water in the CP, this could have introduced bias in derived parameter values. Although the free-water elimination DTI (FWE-DTI) approach has been used widely to distinguish free-water partial-volume effects from tissue’s diffusion in healthy aging and degenerative diseases (83), it has recently been shown that this method is unstable when applied to single-shell DTI data, requiring careful implementation (84). In line with the FEW-DTI approach, a bicomponent relaxometry approach could, in principle, be used to correct for the potential contamination due to CSF in derived T_1_ and T_2_. This model my incorporate a parenchymal compartment and a CSF compartment. However, such approach will require extensive validation and testing which is out of the scope of this work but represents one of the future directions in our CP-related investigations. Further, we note that our derived DTI parameters values could change depending on the choice of the b-values. Several DTI protocols involve use of b-values of 0 and 1000 s/mm^2^ while others incorporate lower b-values. Finally, other factors such as inflammatory markers, diet, and medications were not considered in this work. Indeed, obesity is associated with a plethora of metabolic consequences such as hyperglycemia, insulin resistance and dyslipidemia, while it has recently been shown that the CP is a site of insulin secretion (85). Therefore, the CP may be directly influenced by the state of obesity and the fluid movement differences identified here.

In conclusion, we examined associations between obesity and CP microstructure in a large cohort and across a wide age range of cognitively unimpaired participants. Obesity may represent a modifiable risk factor for disruption of CP structure, and therefore an important therapeutic target. Our results indicate the possibility of a link between obesity and CP structure and volume, and therefore provide a foundation for further investigation of, for example, the effect of diet and physical activity on CP microstructure and function.

## Data availability statement

The raw data supporting the conclusions of this article will be made available by the authors, without undue reservation.

## Ethics statement

The MRI protocol was approved by the MedStar Research Institute and the National Institutes of Health Intramural Ethics Committees, and all examinations were performed in compliance with the standards established by the National Institutes of Health Institutional Review Board. The patients/participants provided their written informed consent to participate in this study.

## Author contributions

JE and MB: research design, results interpretation, and manuscript writing and editing. JA: analysis, results interpretation, and manuscript writing and editing. All authors contributed to the article and approved the submitted version.

## Funding

This work was supported by the Intramural Research Program of the National Institute on Aging of the National Institutes of Health.

## Acknowledgments

We gratefully acknowledge Christopher M. Bergeron, Denise Melvin, and Linda Zukley for their assistance with data acquisition, participant recruitment, and logistics. We also thank all of the study participants.

## Conflict of interest

The authors declare that the research was conducted in the absence of any commercial or financial relationships that could be construed as a potential conflict of interest.

## Publisher’s note

All claims expressed in this article are solely those of the authors and do not necessarily represent those of their affiliated organizations, or those of the publisher, the editors and the reviewers. Any product that may be evaluated in this article, or claim that may be made by its manufacturer, is not guaranteed or endorsed by the publisher.

## Footnotes

1
https://surfer.nmr.mgh.harvard.edu/
